# Draft Genome Sequence of *Geobacter pelophilus* Strain Dfr2, a Ferric Iron–Reducing Bacterium

**DOI:** 10.1128/genomeA.00537-17

**Published:** 2017-06-15

**Authors:** Tomo Aoyagi, Hideaki Koike, Tomotake Morita, Yuya Sato, Hiroshi Habe, Tomoyuki Hori

**Affiliations:** a Environmental Management Research Institute, National Institute of Advanced Industrial Science and Technology (AIST), Tsukuba, Ibaraki, Japan; b Reserch Center for Creative Partnerships, Ishinomaki Senshu University, Ishinomaki, Miyagi, Japan; c Bioproduction Research Institute, National Institute of Advanced Industrial Science and Technology (AIST), Tsukuba, Ibaraki, Japan; d Research Institute for Innovation in Sustainable Chemistry, National Institute of Advanced Industrial Science and Technology (AIST), Tsukuba, Ibaraki, Japan

## Abstract

Here, we report a draft genome sequence of *Geobacter pelophilus* strain Dfr2, a ferric iron–reducing bacterium. This genome information will further our understanding of the mechanisms underlying electron transfer from microorganisms to ferric iron oxides.

## GENOME ANNOUNCEMENT

Microbial ferric iron [Fe(III)] reduction is one of the most important biogeochemical reactions in anoxic natural environments. *Geobacter* spp. belonging to the class *Deltaproteobacteria* have often been identified as the microorganisms involved in the terminal electron-accepting processes in soil, sediment, and groundwater (1, 2). Isolates of *Geobacter* spp. have been classified into a wide variety of phylogenetic clades (3, 4). Moreover, some *Geobacter* spp. can reduce not only soluble forms of Fe(III) but also poorly crystalline Fe(III) oxides such as ferrihydrite (3, 4). Previously, we isolated five species of the genus *Geobacter* from various natural environments using highly crystalline Fe(III) oxide (i.e., goethite, lepidocrocite, hematite, or magnetite) as the electron acceptor (2). However, mainly due to limited genome information, it is still unclear as to which mechanisms underlie the transfer of electrons from *Geobacter* spp. to solid-state iron oxides. *G. pelophilus* is known as one of the representatives of poorly crystalline Fe(III) oxide–reducing bacteria, but its genome sequence has not yet been reported. Here, we report a draft genome sequence of *G. pelophilus* strain Dfr2, which was originally isolated by Straub et al. from freshwater mud in Germany (5).

Strain Dfr2 (DSM12255) was obtained from the Deutsche Sammlung von Mikroorganismen und Zellkulturen (DSMZ) culture collection, and it was grown anaerobically with ferric nitrilotriacetic acid [Fe(III)-NTA] as the electron acceptor. DNA was extracted by a phenol extraction method with chemical cell lysis. A paired-end DNA library (insert size: ~500 bp) and a mate-pair library (insert size: ~4,000 bp) were generated as previously described (6). These two libraries were sequenced using the Illumina MiSeq platform. The paired-end and mate-pair libraries generated 1,871,880 reads (250-bp paired-end) and 1,815,980 reads (250-bp paired-end), respectively. Sequence assembly was performed using the ALLPATHS-LG assembler version 46449 (7), producing 13 contigs with 96.1× and 100.1× genome coverages for the paired-end and mate-pair libraries, respectively. The assembly comprised two scaffolds in total. The largest scaffold length was 4,176,986 bp and covered 96.3% of the total assembled genome sequences. The draft genome of strain Dfr2 was 4,337,996 bp with a G+C content of 61.03%.

A total of 51 tRNA-encoding and 7 rRNA-encoding genes were identified using tRNAscan-SE-version 1.3.1 and RNAmmer version 1.2, respectively (8, 9). The draft genome sequence was annotated using NCBI BLAST version 2.2.29 (BLASTp) with the RefSeq database (10, 11), yielding a total of 3,870 protein-coding sequences. It has been reported that several *Geobacter* spp. have a number of genes coding for *c*-type cytochromes, which are essential for electron transfer. The draft genome of strain Dfr2 contains at least 71 genes coding for the *c*-type cytochromes. Moreover, in the draft genome of strain Dfr2, a type IV pili gene set was also found. Some Fe(III)-reducing *Geobacter* spp. are able to produce the type IV pili, the so-called microbial nanowire, which directly transports electrons to solid-state Fe(III) oxides (12, 13). The draft genome sequence of *G. pelophilus* strain Dfr2 will further an understanding of the mechanisms underlying the transfer of electrons from microorganisms to solid-state Fe(III) oxides.

### Accession number(s).

The *G. pelophilus* Drf2 draft genome sequences have been deposited as two scaffolds in DDBJ/EMBL/GenBank under the accession numbers BDQG01000001 and BDQG01000002. The versions described in this paper are the first versions.
